# Long Noncoding RNA Lnc-MTPAP-1 Overexpressed by Particulate Matter Suppresses Apoptosis in Non-Small Cell Lung Cancer (NSCLC) Cells

**DOI:** 10.3390/ijms262110486

**Published:** 2025-10-28

**Authors:** Ji Won Park, Daeun Kang, Min Hyeok Lee, Yeonwoo Lee, Su Yel Lee, Sin Yung Woo, Keum-Jin Yang, In Beom Jeong, Hee Sun Park, Ji Woong Son, Sun Jung Kwon

**Affiliations:** 1 Division of Pulmonology, Department of Internal Medicine, Daejeon St. Mary’s Hospital, College of Medicine, The Catholic University of Korea, Daejeon 16247, Republic of Korea; herejw@hanmail.net; 2 Division of Pulmonology, Department of Internal Medicine, Konyang University Hospital, College of Medicine, Konyang University, Daejeon 35365, Republic of Korea; kde1324@naver.com (D.K.); spacetravel@naver.com (M.H.L.); sharpion@kyuh.ac.kr (I.B.J.); 3 Myunggok Medical Research Institute, College of Medicine, Konyang University, Daejeon 35365, Republic of Korea; kigasuke@gmail.com (Y.L.); midanf@hanmail.net (S.Y.L.); 4 Division of Endocrinology, Department of Internal Medicine, Konyang University Hospital, Daejeon 35365, Republic of Korea; sywoo1416@gmail.com; 5 Clinical Research Institute, Daejeon St. Mary’s Hospital, Daejeon 16247, Republic of Korea; nadia@cnu.ac.kr; 6 Division of Pulmonology and Allergy, Chungnam National University School of Medicine, Daejeon 35015, Republic of Korea; sparkylove@cnuh.co.kr

**Keywords:** lung cancer, long non-coding RNA (lncRNA), lnc-MTPAP-1, particulate matter (PM10), apoptosis, gene expression, siRNA knockdown, next-generation sequencing (NGS)

## Abstract

Lung cancer remains one of the most common and lethal malignancies worldwide, with poor prognosis largely due to late-stage diagnosis and resistance to therapy. Emerging evidence indicates that long non-coding RNAs (lncRNAs) play critical roles in cancer development, metastasis, and treatment resistance. Particulate matter (PM), a major environmental pollutant and recognized Group 1 carcinogen, has been linked to lung cancer through mechanisms that may involve dysregulation of lncRNA expression. This study aimed to identify PM-responsive lncRNAs in lung cancer, and investigate their potential functional roles. Microarray analysis of lung cancer cell lines A549, H358, H292, and HCC827, exposed to PM10, revealed significant up-regulation of lnc-MTPAP-1. TUNEL staining confirmed that silencing of lnc-MTPAP-1 via siRNA resulted in increased apoptosis across all tested lines. Transcriptome analysis using next-generation sequencing showed that knockdown of lnc-MTPAP-1 altered the expression of apoptosis-related genes, with up-regulation of TNS4, MyD88, and IL6R, and down-regulation of CLPTM1L and EI24. These findings suggest that lnc-MTPAP-1 may exert anti-apoptotic effects in lung cancer cells, and be involved in pollution-induced cancer progression. Further research should explore the therapeutic potential of targeting lnc-MTPAP-1, and better understand the molecular impact of PM exposure on lung cancer pathogenesis.

## 1. Introduction

Lung cancer is the most commonly diagnosed cancer worldwide, and remains a leading cause of cancer-related mortality in both men and women [1]. Late-stage diagnosis and limited therapeutic response contribute to persistently poor cure and survival rates [2]. While advances in targeted therapies and immunotherapy have improved the outcomes for some patients [3,4], their effectiveness is restricted by patient eligibility and the emergence of drug resistance. Therefore, there is an urgent need to overcome resistance mechanisms and identify new therapeutic targets.

With the advent of high-throughput sequencing, it has become evident that only approximately 1.5% of the human genome encodes proteins, whereas the remaining vast majority is transcribed into non-coding RNAs (ncRNAs) [5,6]. Initially considered non-functional, ncRNAs have are now been recognized as key regulators of gene expression and cellular processes [2,6]. Among them, long non-coding RNAs (lncRNAs), defined as transcripts longer than 200 nucleotides, are capable of modulating gene expression at multiple levels, through diverse mechanisms [5,7,8]. LncRNAs are implicated in biological processes, such as apoptosis, differentiation, inflammation, and immune regulation [9], while aberrant lncRNA expression has been associated with a range of diseases, notably cancer [4,10]. LncRNAs are being increasingly studied as potential diagnostic biomarkers, therapeutic targets, and prognostic indicators.

Particulate matter (PM), a major component of air pollution, has been classified by the World Health Organization as a Group 1 carcinogen, and is recognized as a significant risk factor for lung cancer [11]. Recent studies suggest that PM exposure can enhance proliferation, metastasis, and tumorigenicity in both normal lung cells, and in established lung cancer cells [12,13]. While the underlying mechanisms remain unclear, accumulating evidence indicates that PM-induced alterations in lncRNA expression contribute to malignant transformation and progression [14]. Given the increasing contribution of air pollution to the lung cancer burden, lncRNAs affected by PM exposure may represent critical molecular links, and serve as novel therapeutic or prognostic tools.

This study aimed to identify lncRNAs that are responsive to PM exposure in lung cancer cells, and to explore their biological functions. Lung cancer is generally classified into two major histological categories: non-small cell lung cancer (NSCLC, accounting for ~85% of cases) and small cell lung cancer (SCLC, accounting for ~15%) [15]. NSCLC includes adenocarcinoma, squamous cell carcinoma, and large cell carcinoma. SCLC is a highly aggressive tumor type strongly associated with tobacco exposure. Recent studies have reported that exposure to particulate matter is more strongly associated with the initiation and progression of NSCLC than SCLC [12,16]. Although limited epidemiological evidence suggests a possible relationship between PM2.5 exposure and SCLC incidence [17], experimental data on SCLC are scarce, underscoring the need for further research. Accordingly, our experiments were designed using NSCLC cell lines (A549, H358, H292, and HCC827) [12,15]. Through targeted experimental approaches, we sought to uncover lncRNAs involved in apoptosis regulation, and their potential role in cancer progression.

## 2. Results

### 2.1. Lnc-MTPAP-1 Is Overexpressed in Lung Cancer Cell Lines After PM10 Exposure

**Figure 1:**
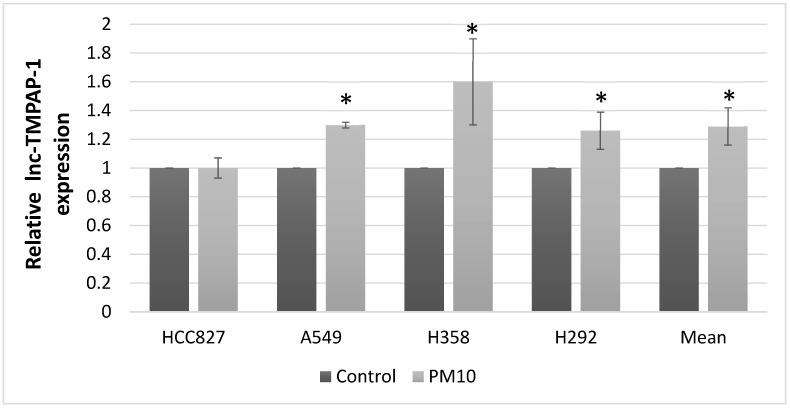
PCR results for relative lncRNA expression differences between PM10-exposed and control groups. Expression changes were validated by PCR for selected lncRNAs identified from Agilent microarray analysis. Among the tested candidates, only lnc-MTPAP-1 exhibited consistent expression patterns between microarray and PCR results. Results for other lncRNAs are available in Supplementary Table S1 (* *p* < 0.05).

We performed Agilent microarray analysis to identify lncRNAs that exhibited altered expression in lung cancer cells following PM10 exposure. Table 1 shows the top five up-regulated and down-regulated lncRNA genes with more than two-fold changes after PM10 treatment in lung cancer cell lines, compared to the control group without PM10 treatment. To verify the lncRNAs identified by microarray, we performed qRT−PCR for each lncRNA in the PM10-treatment group and the control group to compare their differences in expression. Only lnc-MTPAP-1 showed consistent results between the Agilent microarray and qRT−PCR results, whereas the other lncRNAs showed inconsistent results (Figure 1; results for other lncRNAs are available in Supplementary Table S1). Based on the microarray and qRT−PCR, we found that after exposure to PM10, lnc-MTPAP-1 was overexpressed in lung cancer cells. Our microarray data are available as a supplementary file (Supplementary File S1).

### 2.2. Screening for Function of Lnc-MTPAP-1 Using Mini-FACS

**Figure 2:**
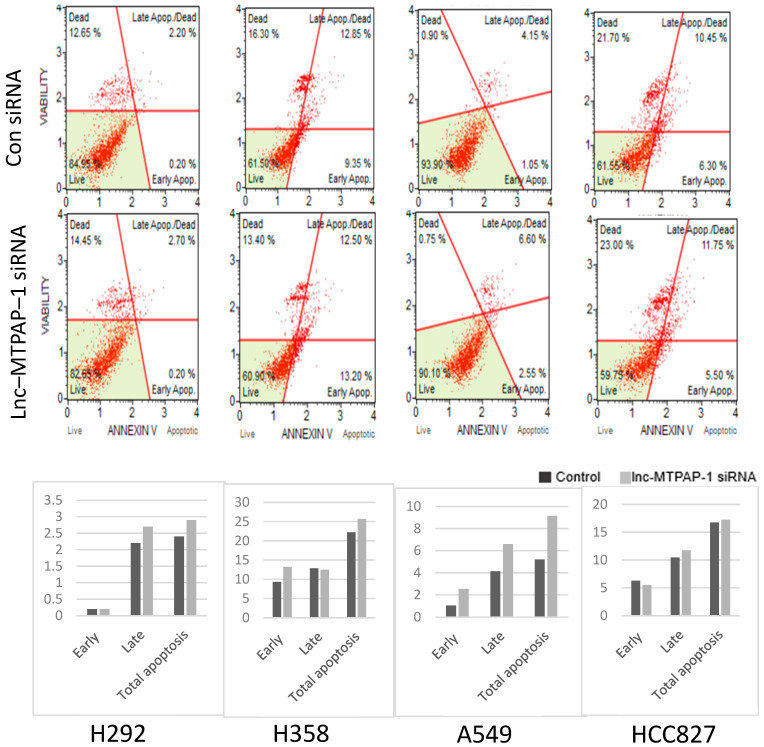
Annexin V & Dead cell (Mini-FACS) results in each cell line. Annexin V/dead cell (mini-FACS) was performed to compare the difference in apoptosis between lnc-MTPAP-1 siRNA-treated and control siRNA-treated groups in each cell line. In all cell lines, there was a trend towards increased apoptosis in the lnc-MTPAP-1 siRNA group. Data are from single mini-FACS experiments (*n* = 1 per cell line) performed as an initial screening; therefore, statistical analysis was not applicable. However, the differences were not statistically significant.

Since lncRNAs are known to be involved in various biological processes, we conducted experiments to determine the biological processes involving lnc-MTPAP-1. As apoptosis is the main mechanism of programmed cell death, we performed annexin V and dead cell experiments using mini-FACS to determine the difference in apoptosis depending on lnc-MTPAP-1 knockdown in each cell line. The results showed that the lnc-MTPAP-1 siRNA treatment group showed a trend towards increased apoptosis, compared to the scrambled siRNA treatment group (Figure 2). 

### 2.3. Lnc-MTPAP-1 Knockdown Induces Apoptosis in Lung Cancer Cell Lines

**Figure 3:**
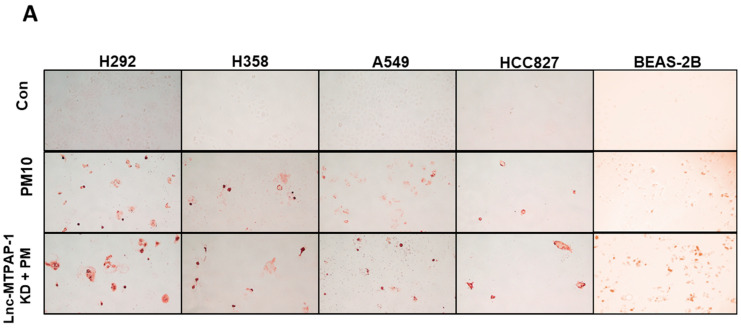

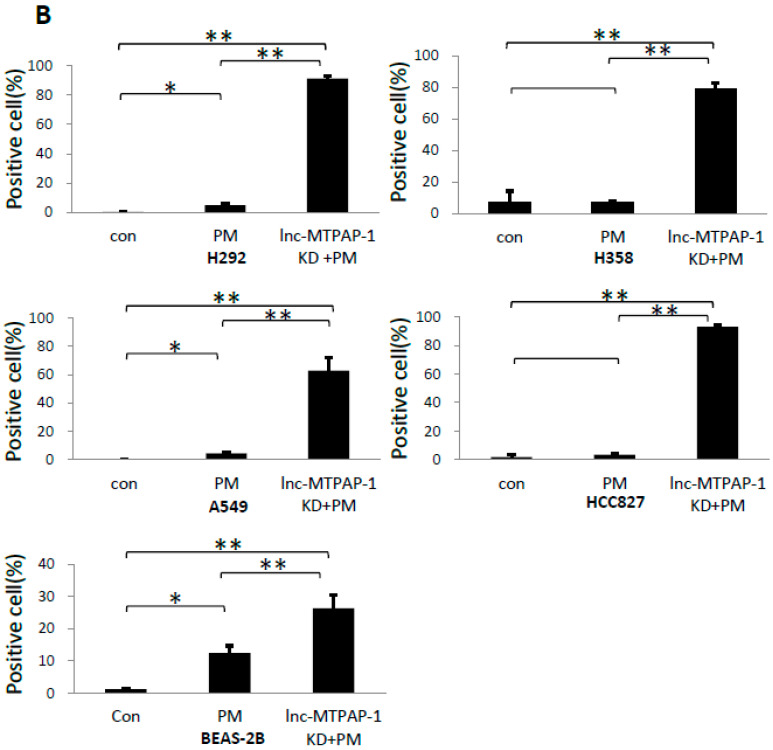
Quantitative analysis of apoptosis by TUNEL assay in lung cancer and non-cancerous BEAS-2B cells. Four lung cancer cell lines (A549, H358, H292, and HCC827) and the non-cancerous bronchial epithelial cell line BEAS-2B were divided into three groups: siRNA control group, siRNA control with PM10 exposure group, and siRNA targeting lnc-MTPAP-1 with PM10 exposure group. TUNEL assay was performed to assess apoptosis. The lnc-MTPAP-1 siRNA-treated group showed a significant increase in apoptosis in all tested cell lines, including BEAS-2B, compared with the control siRNA-treated group. (**A**) Representative TUNEL staining images of apoptotic cells in lung cancer cell lines and BEAS-2B cells. (**B**) Quantitative analysis of TUNEL-positive cells (%). Data are presented as mean ± SD. * *p* < 0.05, ** *p* < 0.001.

To confirm that lnc-MTPAP-1 knockdown caused apoptosis, we performed a TUNEL assay. Each cell line was divided into three groups: (1) control group transfected with scrambled siRNA, (2) PM10-exposed group transfected with scrambled siRNA, and (3) PM10-exposed group transfected with siRNA targeting lnc-MTPAP-1. TUNEL analysis showed that compared to the scrambled siRNA group, the lnc-MTPAP-1 siRNA-treated group revealed a significant increase in apoptosis in all cell lines (Figure 3).

### 2.4. Search for Target Gene of Lnc-MTPAP-1 Using Next Generation Sequencing (NGS)

**Figure 4:**
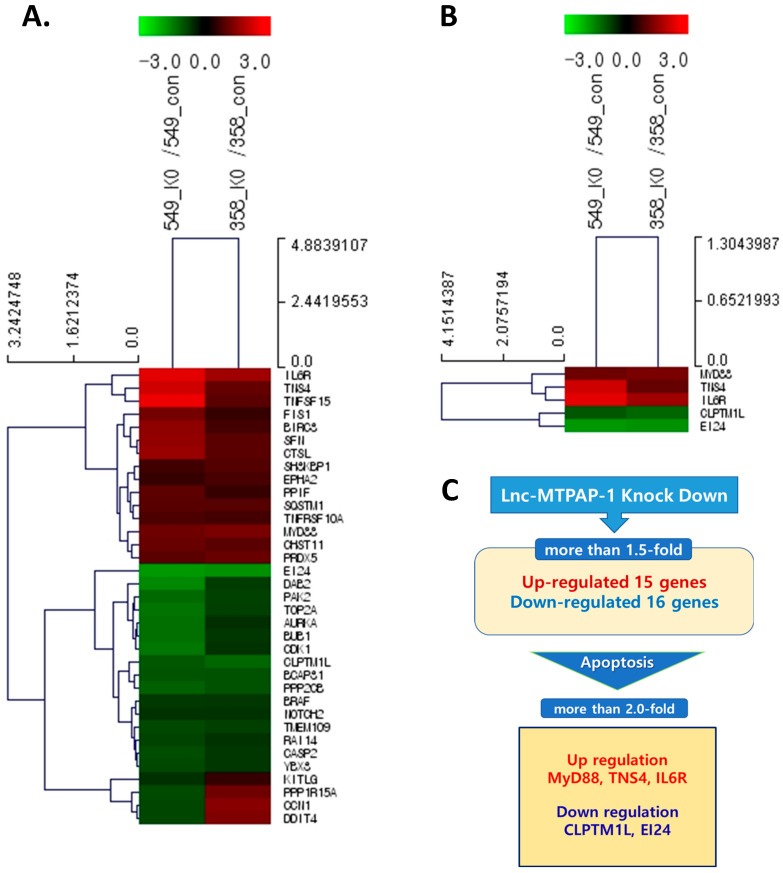
Differential expression of genes by lnc-MTPAP-1 knockdown from the next-generation sequencing analysis. Heat map and hierarchical clustering: Red indicates high expression, while green indicates low relative expression. (**A**) Genes significantly changed more than 1.5-fold. (**B**) and (**C**) Genes changed more than 2-fold that are involved in apoptosis.

**Figure 5:**
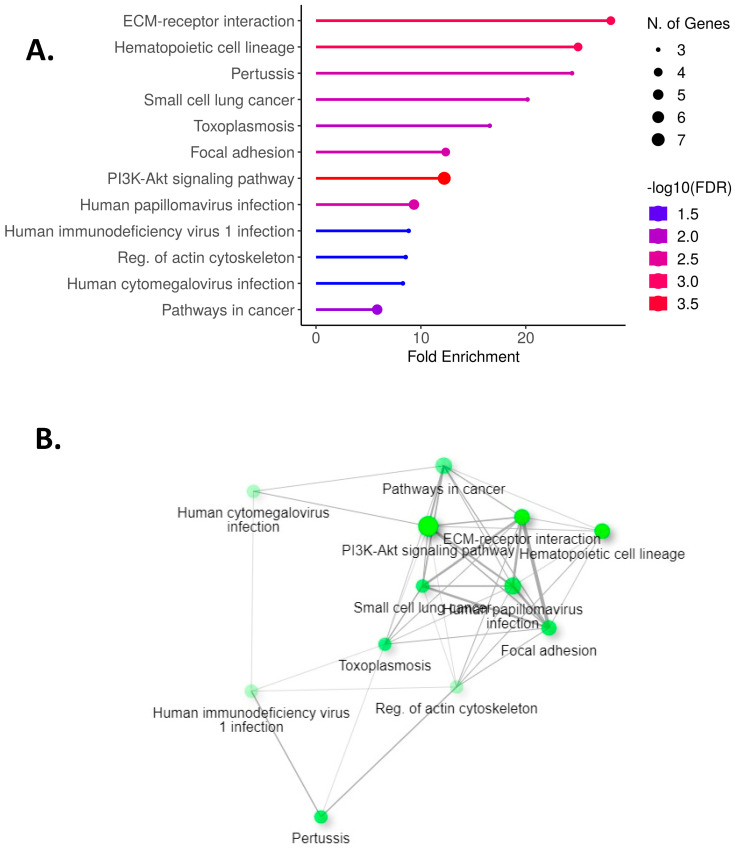
**Pathway enrichment analysis of genes altered by lnc-MTPAP-1 knockdown.** (**A**) KEGG pathway enrichment analysis of differentially expressed genes (fold change > 2) identified by NGS. The color scale indicates statistical significance (−log10 [FDR]), and the dot size represents the number of genes per pathway. (**B**) Network map illustrating the interrelationships among significantly enriched pathways. The node size reflects the number of associated genes, and edge thickness indicates the degree of functional overlap between pathways.

We performed NGS to identify the genes that might interact directly or indirectly with lnc-MTPAP-1. After treating each cell line with PM10, we performed NGS for the group with or without knockdown of lnc-MTPAP-1 to determine the difference in gene expression. Genes exhibiting a simultaneous fold change greater than 1.5 were selected. The results showed that 15 genes were up-regulated, while 16 genes were down-regulated. Among them, genes reported to be involved in apoptosis were selected as target genes for lnc-MTPAP-1. As a result, TNS4, MyD88, and IL6R were up-regulated, whereas CLPTM1L and EI24 were down-regulated by more than twofold (Figure 4). Our NGS data are available as a supplementary file (Supplementary File S2). We performed pathway analysis to explore the biological pathways involved in genes with more than twofold significant expression differences in NGS results (Figure 5). They were analyzed to be involved in ‘ECM-receptor interaction’, ‘small cell lung cancer’, ‘PI3K-Akt signaling pathway’, etc.

### 2.5. Validation of the Relative Expression of Apoptosis-Related Target Genes of Lnc-MTPAP-1

**Figure 6:**
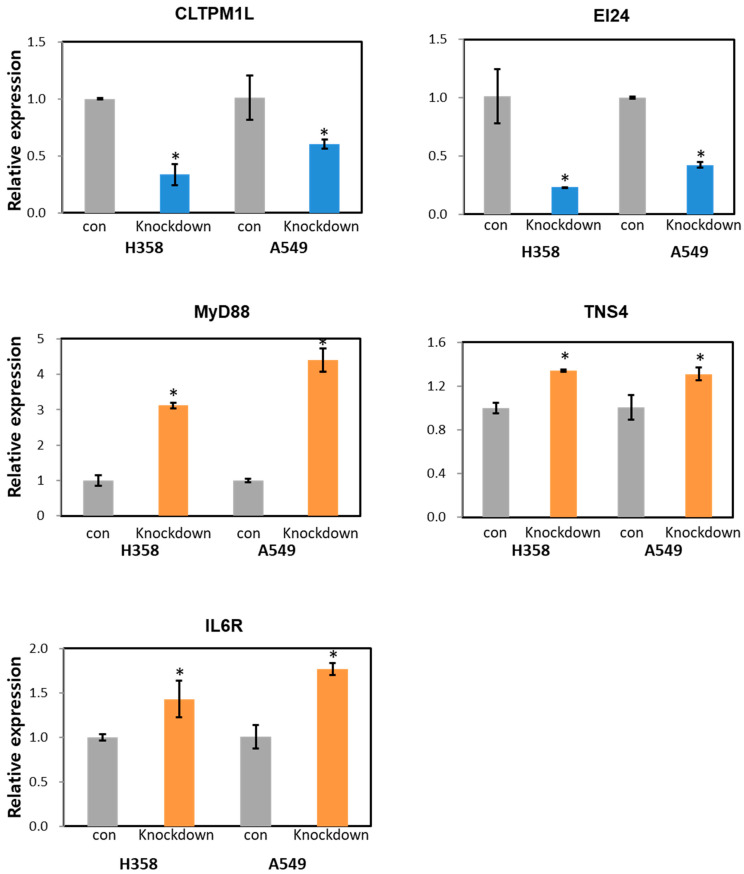
The relative expression level of target genes by qRT−PCR. The relative expression level of target genes of lnc-MTPAP-1 identified from NGS results was verified by qRT−PCR. All genes showed significant differences in expression depending on whether lnc-MTPAP-1 was knocked down (* *p* < 0.05). (con: control group, Knockdown: Lnc-MTPAP-1 siRNA treatment group).

To validate the NGS data, we performed qRT−PCR for the target genes of lnc-MTPAP-1 that are related to apoptosis. As a result, all five up-regulated and down-regulated genes showed consistent expression patterns with the NGS results, depending on whether lnc-MTPAP-1 was knocked down (Figure 6).

## 3. Discussion

In this study, we discovered lnc-MTPAP-1, a lncRNA that changes in response to PM10 stimulation in lung cancer cell lines. We demonstrated that lnc-MTPAP-1 knock-down by siRNA increased apoptosis in lung cancer cell lines, indicating that the lncRNA suppresses apoptosis in lung cancer cells. We also identified a list of target genes expected to be involved in the action of lnc-MTPAP-1 using NGS.

Approximately 99% of the world’s population lives in places that do not meet WHO air quality guideline levels [18]. Particulate matter is one of the major public health concerns, affecting a variety of diseases in multiple organs beyond lung disease. PM10 refers to inhalable particles with a diameter of less than 10 μm containing metals, pollens, organic compound, inorganic ions, etc. [19]. PM10 is mainly retained in the mucus layer. Although it can be easily cleared from the airways, some of its components can remain in the airways, and induce inflammation and oxidative stress [19]. As a result, PM10 can cause airway inflammatory disease, worsen existing airway disease, and even affect the development of cancer [20,21]. Furthermore, exposure to PM10 drives cancer invasion and metastasis through epithelial mesenchymal transition (EMT) [22], or dysregulation of lncRNAs [23,24]. Miguel et al. demonstrated that PM10 up-regulates lncRNA NORAD, impairing the spindle assembly checkpoint (SAC) in A549 lung cells. They suggested that PM10 exposure promotes genomic instability and cell survival in lung cancer cells [23]. Biao Yang et al. showed that lung cancer cells (A549, H1299) exposed to PM2.5 had higher cell proliferation and viability and increased migration, compared to the unexposed group [13]. They also identified genes whose expression was altered in PM2.5-exposed lung cancer cells, and demonstrated that these genes play an important role in the proliferation and survival of tumor cells [13]. Our study found that the expression of the LncRNA lnc-MTPAP-1 was increased in lung cancer cells exposed to PM10, and knock-down of this increased apoptosis. These results suggest that lnc-MTPAP-1 is involved in suppressing apoptosis of lung cancer cells in response to external stimulus PM10, suggesting that it is involved in lung cancer survival. However, it is unclear whether it is associated with disease progression, or whether this is to maintain cellular homeostasis in response to the toxicity of PM10. Futher research is needed to confirm its role. Notably, similar effects were observed in BEAS-2B cells, suggesting that lnc-MTPAP-1 may act as a general protective factor in normal epithelial cells, whereas in malignant cells such mechanisms may contribute to persistence.

**Figure 7:**
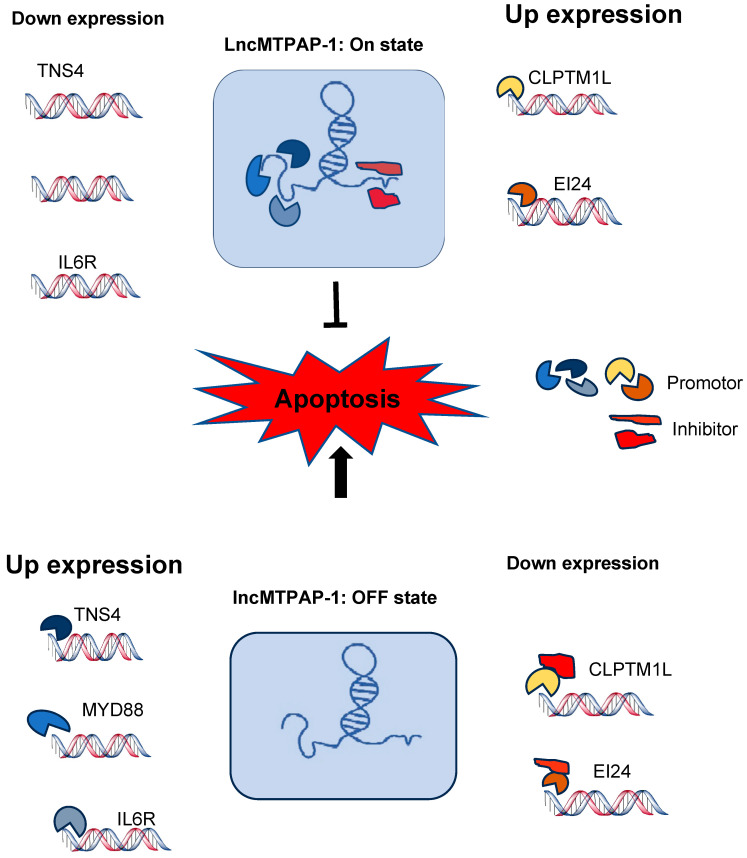
Schematic of the inferred mechanism by which Lnc-MTPAP-1 acts on up- and down-regulated genes. Knockdown of lnc-MTPAP-1 results in the down-regulation of the CLPTM1L, and EI24 genes, and the up-regulation of the TNS4, MyD88, and IL6R genes. Lnc-MTPAP-1 is believed to regulate genes that increase and decrease cell death. Although Lnc-MTPAP-1 knockdown increased cell death, the direction of target gene expression did not match what was expected, based on its previously reported function. This discrepancy may be explained by long non-coding RNAs (lncRNAs) acting through multiple mechanisms, one of which is acting as a decoy. In other words, Lnc-MTPAP-1 is thought to regulate target gene expression by acting as a sponge for transcriptional promoters or repressors.

Using NGS, we identified genes altered by lnc-MTPAP-1 knockdown, and selected genes that showed a significant change of more than 2-fold. CLPTM1L and EI24 were down-regulated, whereas TNS4, MyD88, and IL6R were up-regulated. CLPTM1L is overexpressed in several cancers, including lung cancer, renal cell carcinoma, and laryngeal squamous cell carcinoma. It has been reported to be associated with anti-apoptotic mechanisms and drug resistance [25]. EI24 is related to ER stress-induced apoptosis. EI24 depletion can enhance ER stress-induced apoptosis [26]. On the other hand, EI24 is involved in the activation of autophagy and the suppression of cell growth. EI24 can act as a tumor suppressor gene. Down-modulation of EI24 is associated with drug resistance and poor prognosis [27]. The IL6R gene is also known to regulate cell growth and differentiation. It is involved in immune response and inflammation [28]. As well, accumulating studies have shown that IL6R is associated with several tumors, and is involved in tumor angiogenesis, metastasis, and invasion [28,29]. MyD88 is known as a central hub in immune and inflammatory responses [30]. Several studies have shown that MyD88 and MyD88-related signaling are involved in carcinogenesis and progression [31]. TNS4 is a member of the tensin gene family. Tensins are known to play important roles in cell adhesion, migration, invasion, apoptosis, and proliferation [32]. Aberrant expression of TNS4 has been reported in several cancers, including breast, colon, gastric, and lung cancers [33]. Genes altered by lnc-MTPAP-1 knock-down are involved in various biological processes, such as cancer progression, metastasis, angiogenesis, and migration, or inflammatory and immune responses. Functional analysis of these genes further suggests that the function of lnc-MTPAP-1 may be closely related to the development and progression of cancer, or to immune and inflammatory responses in the cancer microenvironment. Although our data identified several potential downstream genes that may be regulated by lnc-MTPAP-1, we did not perform confirmatory experiments to validate their precise signaling roles in the current study. We acknowledge this limitation and have presented a schematic model (Figure 7) that integrates 24r transcriptomic findings and proposes a possible mechanism. Future research will focus on experimental validation of these candidate targets and downstream signaling circuits to further delineate the molecular mechanism of lnc-MTPAP-1 in NSCLC. 

Several lncRNAs have been reported to be involved in disease development by affecting the apoptosis pathway. The lncRNA TUG1 has been reported to show up-regulated expression in osteosarcoma, and can also inhibit the apoptosis of osteosarcoma cells. One of the mechanisms of action of TUG1 is through the competitive endogenous RNA (ceRNA) of miRNA. TUG1 can directly bind to the 5′-terminus “−CUGACAA−” sequence of MiR−132−3p, and inhibit the expression of MiR−132−3p. As a result, it can suppress osteosarcoma cell death by reducing the activity of caspase 3 in the apoptosis pathway [34]. Growth arrest-specific 5 (Gas5) gene is a lncRNA known to be required for normal growth arrest, slowing the cell cycle, and regulating apoptosis [35]. Expression of the GAS5 has been reported to be reduced in various cancers [36]. Although the function and mechanism of action of the lncRNA GAS5 remain poorly understood, relevant studies have shown that the 3′ terminus structure of GAS5 can act as a glucocorticoid response element (GRE) decoy, competing with DNA−GREs for binding to the glucocorticoid receptor (GR). This renders it sensitive to apoptosis by interfering with the anti-apoptotic action associated with glucocorticoids [35,37]. Ma et al. have shown that lncRNA, which is highly up-regulated in liver cancer (HULC), can protect against TNF-a-induced apoptosis, and that this acts through the regulation of miR−9 expression [38]. Lnc-MTPAP-1 is thought to regulate genes that increase apoptosis, as well as genes that decrease apoptosis. In addition, when Lnc-MTPAP-1 was knocked down, apoptosis increased, but the direction of increase or decrease in the expression of its target genes did not match the direction expected from their previously already reported functions. This may be attributed to the function of lncRNAs through multiple mechanisms. LncRNA regulates gene expression through various mechanisms at multiple levels [39]. LncRNAs can modulate chromosome structure and function by interacting with chromatin modifiers, or recruiting them to target gene promoters to repress or enhance transcription, and they can also affect RNA splicing and translation [39]. Several models have been proposed for how lncRNAs act as regulators, such as signal, enhancer, decoy, scaffold, and guide RNAs [39]. Among these, decoy lncRNAs to regulate transcription by sequestering regulatory factors, such as transcription factors, miRNAs, and subunits of the chromatin modification complex, thereby limiting their action [39]. Although further studies are needed, based on the function of genes in response to lnc-MTPAP-1 knockdown and the direction of up-regulation and down-regulation, it can be inferred that one of the mechanisms of action of lnc-MTPAP-1 is to act as a decoy LncRNA. In other words, lnc-MTPAP-1 is thought to regulate the expression of target genes by acting as a sponge for transcription promoting or repressing factors (Figure 7).

To predict the effect associated with lnc-MTPAP-1, we used a prediction program to identify interacting proteins. We identified the top 10 predicted interactive proteins, and investigated their functions. Most proteins were found to play a regulatory role in relation to mRNA. One of these proteins, TIA−1 (T cell intracellular antigen-1), has been reported to be an apoptosis-promoting factor that regulates the alternative splicing of several pre-mRNAs [39,40] (Supplementary Table S2). 

This study is significant, as it is the first to identify lnc-MTPAP-1, a lncRNA that modulates apoptosis in lung cancer cells in response to PM10, a major carcinogen, thereby providing some mechanisms for how air pollution contributes to the survival or progression of lung cancer cells. Also, our findings suggest that lnc-MTPAP-1 plays an anti-apoptotic role in lung cancer cells, which may contribute to cancer progression. Targeting lnc-MTPAP-1 may provide new therapeutic opportunities for lung cancer treatment.

This study has several limitations. First, the study was conducted in lung cancer cell lines (A549, H358, H292, and HCC827) under controlled conditions that may not fully replicate the complex tumor microenvironment or in vivo interactions. Second, owing to the length of the gene, we were unable to assess the effect of lnc-MTPAP-1 overexpression. In addition, since this study focused on apoptosis, the function identified in this study might comprise only a small portion of the functions of lnc-MTPAP-1. Our study only demonstrated changes in target gene expression following knockdown of lnc-MTPAP-1, without directly confirming the interactions between lnc-MTPAP-1 and regulatory elements.

## 4. Materials and Methods

### 4.1. Cell Culture and PM10 Treatment

Four human lung cancer cell lines (A549, H358, H292, HCC827) and one human normal epithelial cell line (BEAS-2B) were obtained from the American Type Culture Collection (ATCC, Manassas, VA, USA). We cultured the A549, H358, H292 and HCC827 Cells in RPMI−1640 medium (Gibco, Invitrogen, Grand Island, New York, NY, USA) and BEAS 2B in a keratinocyte serum-free medium, supplemented with 10% fetal bovine serum (FBS; Gibco, Invitrogen, Carlsbad, CA, USA), 100 U/mL penicillin/streptomycin, and 0.25 μg/mL amphotericin B, and incubated at 37 °C in 5% CO_2_. The cells were passaged every three days. For particulate matter exposure, ERM^®^ CZ120 PM10 (Sigma-Aldrich, Warsaw, Poland) was suspended in phosphate-buffered saline, and applied at 200 μg/mL for 24 h (the full composition is provided in the Supplementary Table S3).

### 4.2. Microarray Analysis

#### 4.2.1. Amplification and Hybridization

Each cell line was divided into PM10-treated and untreated groups. Total RNA was amplified and labeled with Cyanine 3 using Agilent’s Low Input RNA Amplification Kit PLUS. Hybridization was performed with Agilent’s Gene Expression Hybridization Kit. Labeled cDNAs were washed using Agilent’s gene expression wash buffer.

#### 4.2.2. Signal Analysis

lncRNA expression was analyzed using Agilent’s GeneSpring GX chip V3 and Agilent’s GeneSpring software V14.9. A minimum threshold of 5.0 was used to filter noise. Normalization was performed by dividing signal intensities by the 50th percentile of all probes. Fold changes were calculated using a one-channel method (test/control). Probes were excluded if they were flagged because of a low signal or poor reproducibility. lncRNAs showing >1.5-fold consistent changes across all four cell lines were selected.

### 4.3. siRNA-Mediated Knockdown of Lnc-MTPAP-1 in Lung Cancer Cells

#### 4.3.1. Cell Plating and LncMTPAP−1 Gene Knockdown

Five cell lines (A549, H358, H292, HCC827, BEAS -2B) were seeded at a density of 2.5 × 10^5^ cells/mL into each well of a 6-well plate, and incubated at 37 °C with 5% CO_2_ for 20 h. Cells were transfected with lnc-MTPAP-1 siRNA (RNA−ACU CUU GUG UCU CCC UUC A) at a final concentration of 100 nM, using Lipofectamine RNAiMAX (Invitrogen) for 5 h.

#### 4.3.2. RNA Isolation

Total RNA was isolated using Trizol reagent (Invitrogen California, 92008, USA). RNA quality was assessed using a TapeStation 4000 System (Agilent Technologies, Amstelveen, The Netherlands). RNA quantification was performed by ND−2000 spectrophotometry (Thermo Inc., Dover, DE, USA).

#### 4.3.3. qPCR Analysis of LncRNA

To verify the expression of lncMTPAP-1, A549, H358, H292, and HCC827 cells from the two groups (one was treated with PM10, while the other was untreated) were harvested for real-time PCR. Quantitative real-time PCR (qRT−PCR) was performed using a LightCycler 96 (Roche, Rotkreuz, Switzerland). Each experiment included three biological replicates. Cycling conditions were as follows: preincubation at 95 °C for 3 min, followed by 45 cycles of 95 °C for 30 s, 57 °C for 30 s, and 72 °C for 30 s. The melting step was conducted at 95 °C for 10 s, 65 °C for 1 min, and 97 °C for 1 s. Relative mRNA levels were determined using the 2−ΔΔCT method. The primer sequences were as follows: lnc-MTPAP-1 exon sense primer 5′-TTTGGCTCCTCAGTCAACAC-3′, exon antisense primer 5′-GAACACCAAGGCTCTCACTC-3′, lnc-MTPAP-1 intron sense primer 5′-TCTAAGGTAGGTCTTCAACGC-3′, and intron antisense primer 5′-GCGGCCATGTCTTCAATAAG-3′. The qPCR results for the remaining nine lncRNAs are not shown.

### 4.4. Transferase-Mediated dUTP Nick End-Labeling (TUNEL) Staining

To assess apoptosis after transfecting siRNA of LncRNA (MTPAP−1) in A549, H358, H292, HCC827, and BEAS-2B cells, TUNEL staining was performed using an ApopTag Plus Peroxidase In Situ Apoptosis Kit (Millipore, Burlington, MA, USA), according to the manufacturer’s directions. The ratio of positive to total cells was calculated.

### 4.5. Next-Generation Sequencing After Knockdown of Lnc-MTPAP-1

#### 4.5.1. Library Preparation and Sequencing

Libraries were prepared using total RNA and a CORALL RNA−Seq V2 Library Prep Kit (LEXOGEN, Vienna, Austria). Ribosomal RNA (rRNA) was removed using a RIBO COP rRNA depletion kit (LEXOGEN, Inc., Austria). These rRNA-depleted RNAs were used for cDNA synthesis and shearing, following the manufacturer’s instructions. Indexing was performed using Illumina indices 1−12. The enrichment step was conducted using PCR. Subsequently, libraries were assessed using an Agilent 2100 Bioanalyzer with a DNA High Sensitivity Kit to determine the mean fragment size. Quantification was performed using the library quantification kit and a Step One Real-Time PCR System (Life Technologies, Carlsbad, CA, USA). High-throughput sequencing was conducted as paired-end 100 bp reads, using a NovaSeq 6000 (Illumina, Inc, San Diego, CA, USA).

#### 4.5.2. Data Analysis

Quality control of the raw sequencing data was performed using FastQC_V0.12.1. Adapter and low-quality reads were removed using Fastp.0.23.1. Trimmed reads were then mapped to the reference genome using STAR_2.7.10b. Quantification of reads was performed using Salmon V1.10. Read counts were processed based on TMM + CPM normalization method using EdgeR 3.42.4. Data mining and graphic visualization were performed using ExDEGA5,2,12 (Ebiogen Inc., Seoul, Korea). Genes in the group of A549 and H358 cells with lnc-MTPAP-1 knockdown compared to the control are summarized by cell line with a fold change ≥ 1.5, normalized value ≥ 4. Fifteen genes were up-regulated, and 16 genes were down-regulated.

### 4.6. qPCR of mRNA

To confirm the up-regulation or down-regulation of mRNA expression, qPCR was performed. Total RNA was isolated from A549 and H358 cells in the presence or absence of siRNA for lncMTPAP−1 using Trizol reagent (Invitrogen California, 92008, USA) Complementary DNA (cDNA) was synthesized using reverse transcriptase premix (Elpis Biotech, Daejeon, Korea), and amplified using Power SYBR Green polymerase chain reaction master mix (Applied Biosystems, Carlsbad, CA, USA) and gene-specific primer pairs (Table 2 lists the primer sequences). The qPCR was performed as an ABI 7500 FAST instrument (Applied Biosystems, Carlsbad, CA, USA). Relative expression levels were obtained after normalization with GAPDH, compared to the control group.

## 5. Conclusions

The lncRNA identified in this study, lnc-MTPAP-1, increased expression when exposed to PM10 in lung cancer cell lines, and its knockdown led to increased apoptosis. We also identified genes that showed changes in expression upon knockdown of this lncRNA and investigated their functions. We found that they were involved in processes such as progression or suppression, metastasis, invasion, adhesion, and migration, in various cancers. We also suggest that lnc-MTPAP-1 may play an important role in processes associated with lung cancer, based on the roles of its target genes. Further studies are required to identify a more specific mechanism of action, and determine the significance of the role of lnc-MTPAP-1 in lung cancer and lung disease.

## Figures and Tables

**Table 1:** Top 5 up- and down-regulated LncRNA genes based on microarray results.

**Top 5 Up-Regulated LncRNA Genes**					
**Gene**	${Log}_{2}$ **FC**				**Mean** ${Log}_{2}$ **FC**
	**HCC827**	**A549**	**H358**	**H292**	
lnc-MTPAP-1	1.1412245	1.0393772	6.814241	2.1546037	2.7873616
lnc-PLXND1-1	1.11237	1.2055317	4.872442	2.4244833	2.4037068
lnc-DHX40-1	1.2124051	1.2725444	4.540834	1.8083729	2.2085391
THAP9−AS1	1.1720212	1.8104212	3.9262705	1.0608561	1.9923923
lnc-USP28-1	1.3274853	2.2720706	2.4233804	1.3167702	1.8349266
**Top 5 Down-Regulated LncRNA Genes**					
**Gene**	${Log}_{2}$ **FC**				**Mean** ${Log}_{2}$ **FC**
	**HCC827**	**A549**	**H358**	**H292**	
lnc-TRA2A-1	0.5045274	0.8241876	0.21710351	0.25026	0.4490196
HID1−AS1	0.746688	0.25143218	0.46924052	0.55981	0.5067927
MIR200CHG	0.4364092	0.4170083	0.4236734	0.7643422	0.5103583
lnc-PBX1-2	0.7775569	0.5471765	0.4881028	0.3748579	0.5469235
LOC101929217	0.4969948	0.398749	0.582493	0.7563759	0.5586532

The genes are arranged in order of average value. FC indicates fold change.

**Table 2:** The sequence of qRT−PCR primers.

Gene	Sequence (5′ to 3′)
CLPTM1L	F: AGAGGAGAGCCAGGGATTGA/ R: CCACCTTCTTGTGAGCATCC
EI24	F: GCTGGTGCAGGAAATGAAGA/ R: TGAGTCTGGTAGGCGATGAA
IL6R	F: AAGGTGCGGATGAGTTTGAG/ R: CTTCCACGATGCTGATGTTG
MyD88	F: TGTGTGGTGAAGATGCGTTT/ R: TGGGAAAGGAGAGGTGGAAG
TNS4	F: ATAGAGCGTGGTCCAGTTCG/ R: GGTCCACCTCCGAGTTCTTC
GAPDH	F: CTCCTGCACCACCAACTGCT/ R: GGGCATGGACTGTGGTCATG

## Data Availability

The original contributions presented in this study are included in the article/supplementary material. Further inquiries can be directed to the corresponding authors.

## Supplementary Materials

The following supplementary information can be downloaded at https://www.mdpi.com/article/10.3390/ijms262110486/s1.
