# Supplementary material for: Long Noncoding RNA Lnc-MTPAP-1 Overexpressed by Particulate Matter Suppresses Apoptosis in Non-Small Cell Lung Cancer (NSCLC) Cells
Source: Int J Mol Sci. 2025 Oct 28;26(21):10486. doi: 10.3390/ijms262110486 (PMC12608595; doi:10.3390/ijms262110486)
Supplement: Supplementary file 1 [file ijms-26-10486-s001.zip › ijms-26-10486-s001/Supplementary Data.pptx]

## Slide 1
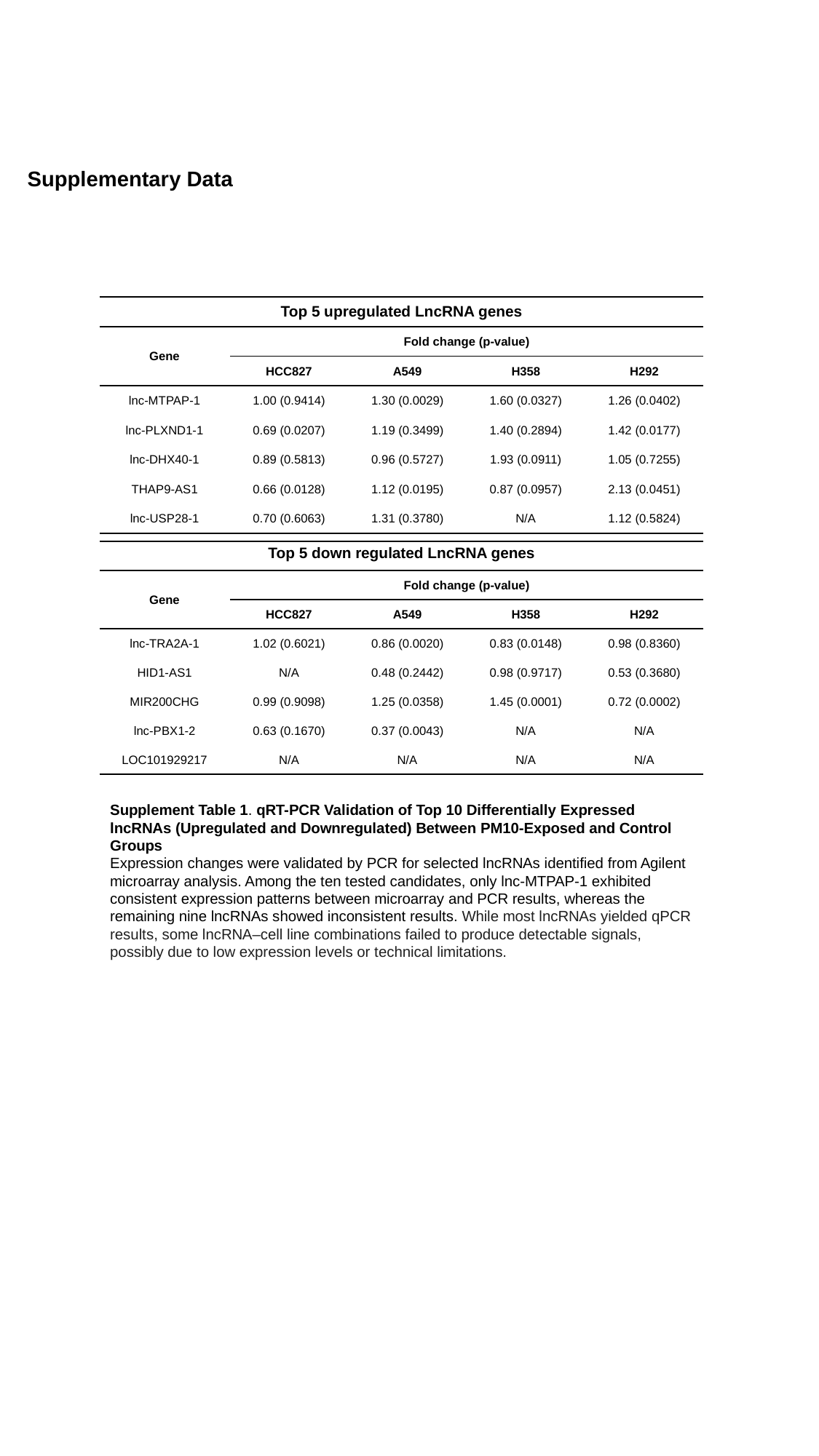

Supplementary Data
| Top 5 upregulated LncRNA genes | | | | |
| --- | --- | --- | --- | --- |
| Gene | Fold change (p-value) | | | |
| | HCC827 | A549 | H358 | H292 |
| lnc-MTPAP-1 | 1.00 (0.9414) | 1.30 (0.0029) | 1.60 (0.0327) | 1.26 (0.0402) |
| lnc-PLXND1-1 | 0.69 (0.0207) | 1.19 (0.3499) | 1.40 (0.2894) | 1.42 (0.0177) |
| lnc-DHX40-1 | 0.89 (0.5813) | 0.96 (0.5727) | 1.93 (0.0911) | 1.05 (0.7255) |
| THAP9-AS1 | 0.66 (0.0128) | 1.12 (0.0195) | 0.87 (0.0957) | 2.13 (0.0451) |
| lnc-USP28-1 | 0.70 (0.6063) | 1.31 (0.3780) | N/A | 1.12 (0.5824) |
| | | | | |
| Top 5 down regulated LncRNA genes | | | | |
| Gene | Fold change (p-value) | | | |
| | HCC827 | A549 | H358 | H292 |
| lnc-TRA2A-1 | 1.02 (0.6021) | 0.86 (0.0020) | 0.83 (0.0148) | 0.98 (0.8360) |
| HID1-AS1 | N/A | 0.48 (0.2442) | 0.98 (0.9717) | 0.53 (0.3680) |
| MIR200CHG | 0.99 (0.9098) | 1.25 (0.0358) | 1.45 (0.0001) | 0.72 (0.0002) |
| lnc-PBX1-2 | 0.63 (0.1670) | 0.37 (0.0043) | N/A | N/A |
| LOC101929217 | N/A | N/A | N/A | N/A |
Supplement Table 1. qRT-PCR Validation of Top 10 Differentially Expressed lncRNAs (Upregulated and Downregulated) Between PM10-Exposed and Control Groups
Expression changes were validated by PCR for selected lncRNAs identified from Agilent microarray analysis. Among the ten tested candidates, only lnc-MTPAP-1 exhibited consistent expression patterns between microarray and PCR results, whereas the remaining nine lncRNAs showed inconsistent results. While most lncRNAs yielded qPCR results, some lncRNA–cell line combinations failed to produce detectable signals, possibly due to low expression levels or technical limitations.

## Slide 2
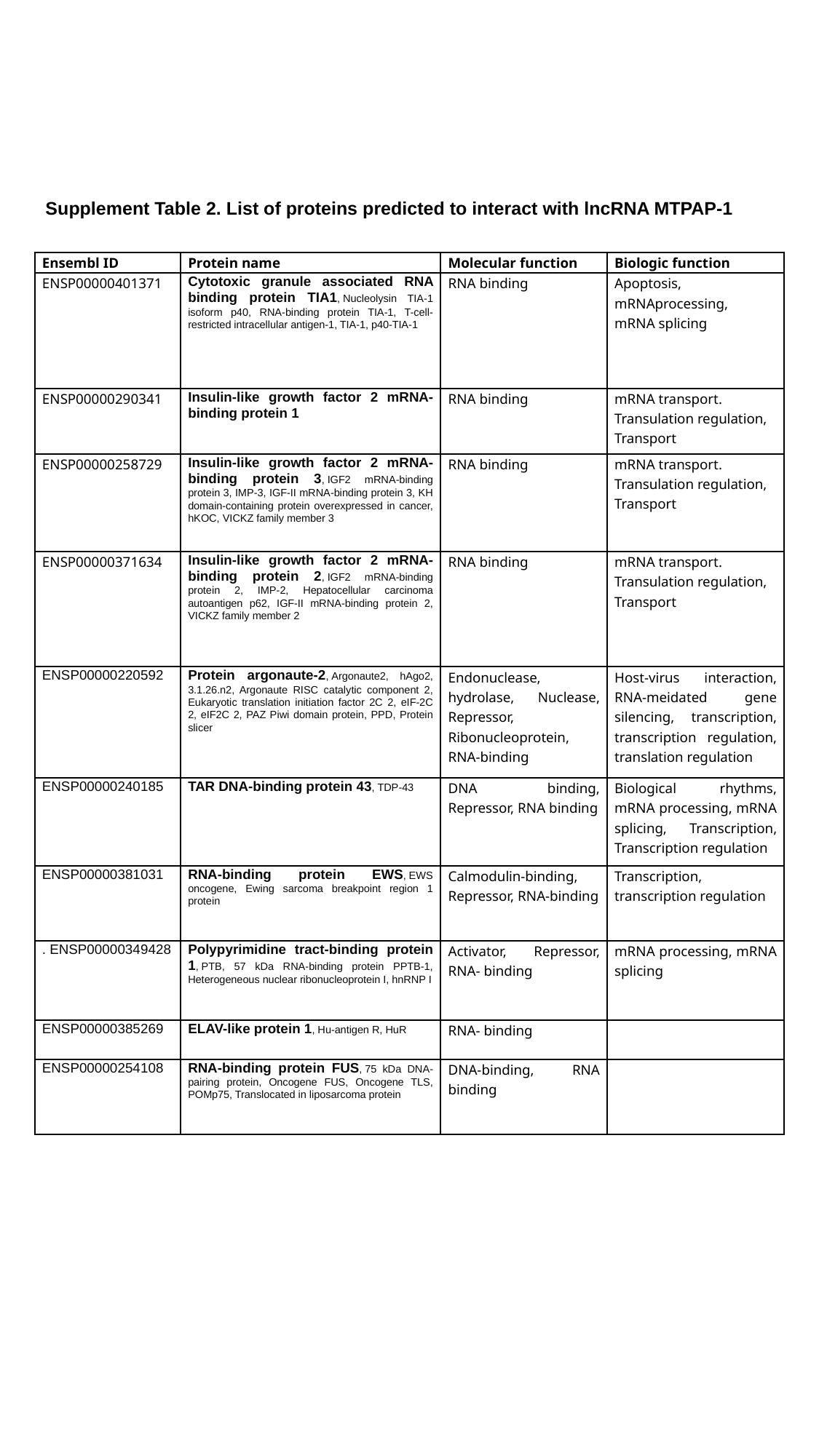

Supplement Table 2. List of proteins predicted to interact with lncRNA MTPAP-1
| Ensembl ID | Protein name | Molecular function | Biologic function |
| --- | --- | --- | --- |
| ENSP00000401371 | Cytotoxic granule associated RNA binding protein TIA1, Nucleolysin TIA-1 isoform p40, RNA-binding protein TIA-1, T-cell-restricted intracellular antigen-1, TIA-1, p40-TIA-1 | RNA binding | Apoptosis, mRNAprocessing, mRNA splicing |
| ENSP00000290341 | Insulin-like growth factor 2 mRNA-binding protein 1 | RNA binding | mRNA transport. Transulation regulation, Transport |
| ENSP00000258729 | Insulin-like growth factor 2 mRNA-binding protein 3, IGF2 mRNA-binding protein 3, IMP-3, IGF-II mRNA-binding protein 3, KH domain-containing protein overexpressed in cancer, hKOC, VICKZ family member 3 | RNA binding | mRNA transport. Transulation regulation, Transport |
| ENSP00000371634 | Insulin-like growth factor 2 mRNA-binding protein 2, IGF2 mRNA-binding protein 2, IMP-2, Hepatocellular carcinoma autoantigen p62, IGF-II mRNA-binding protein 2, VICKZ family member 2 | RNA binding | mRNA transport. Transulation regulation, Transport |
| ENSP00000220592 | Protein argonaute-2, Argonaute2, hAgo2, 3.1.26.n2, Argonaute RISC catalytic component 2, Eukaryotic translation initiation factor 2C 2, eIF-2C 2, eIF2C 2, PAZ Piwi domain protein, PPD, Protein slicer | Endonuclease, hydrolase, Nuclease, Repressor, Ribonucleoprotein, RNA-binding | Host-virus interaction, RNA-meidated gene silencing, transcription, transcription regulation, translation regulation |
| ENSP00000240185 | TAR DNA-binding protein 43, TDP-43 | DNA binding, Repressor, RNA binding | Biological rhythms, mRNA processing, mRNA splicing, Transcription, Transcription regulation |
| ENSP00000381031 | RNA-binding protein EWS, EWS oncogene, Ewing sarcoma breakpoint region 1 protein | Calmodulin-binding, Repressor, RNA-binding | Transcription, transcription regulation |
| . ENSP00000349428 | Polypyrimidine tract-binding protein 1, PTB, 57 kDa RNA-binding protein PPTB-1, Heterogeneous nuclear ribonucleoprotein I, hnRNP I | Activator, Repressor, RNA- binding | mRNA processing, mRNA splicing |
| ENSP00000385269 | ELAV-like protein 1, Hu-antigen R, HuR | RNA- binding | |
| ENSP00000254108 | RNA-binding protein FUS, 75 kDa DNA-pairing protein, Oncogene FUS, Oncogene TLS, POMp75, Translocated in liposarcoma protein | DNA-binding, RNA binding | |

## Slide 3
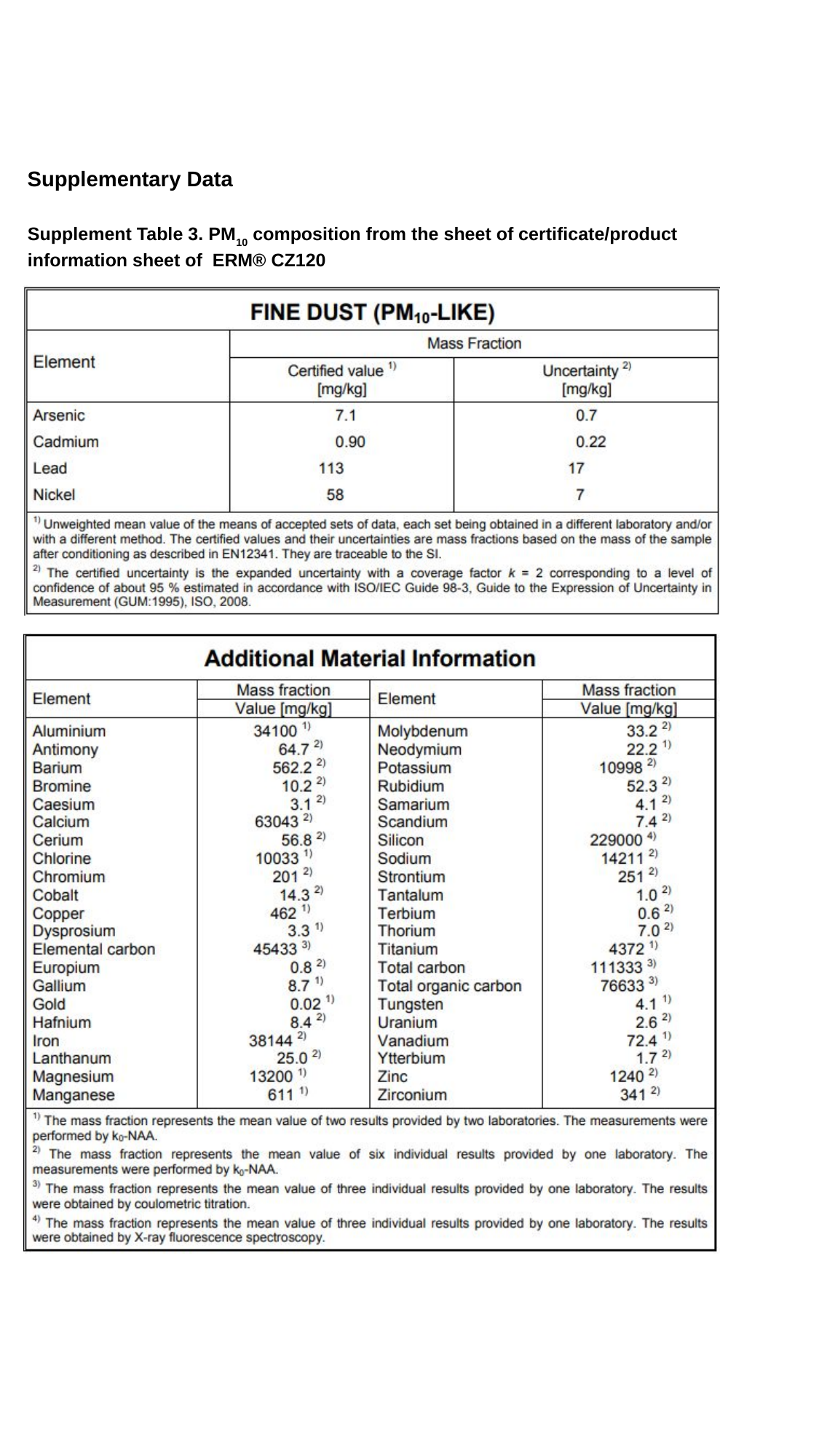

Supplementary Data
Supplement Table 3. PM10 composition from the sheet of certificate/product information sheet of ERM® CZ120
